# Structure and Oxidation Behavior of Multicomponent (Hf,Zr,Ti,Nb,Mo)C Carbide Ceramics

**DOI:** 10.3390/ma16083163

**Published:** 2023-04-17

**Authors:** Elena Mirovaya, Alexander Burlachenko, Nikolay Kulagin, Yuriy Mirovoy, Alexey Neiman, Svetlana Buyakova

**Affiliations:** 1 Institute of Strength Physics and Materials Science SB RAS, 634055 Tomsk, Russia; 2 Engineering School of New Production Technologies, Tomsk Polytechnic University, 634050 Tomsk, Russia

**Keywords:** multicomponent ceramics, high-entropy carbide, high—temperature in situ X-ray, TG-DSC

## Abstract

Multicomponent ceramics based on transition metals carbides are widely known for their excellent physicomechanical properties and thermal stability. The variation of the elemental composition of multicomponent ceramics provides the required properties. The present study examined the structure and oxidation behavior of (Hf,Zr,Ti,Nb,Mo)C ceramics. Single-phase ceramic solid solution (Hf,Zr,Ti,Nb,Mo)C with FCC structure was obtained by sintering under pressure. It is shown that during the mechanical processing of an equimolar powder mixture of TiC–ZrC–NbC–HfC–Mo_2_C carbides, the formation of double and triple solid solutions occurs. The hardness of (Hf,Zr,Ti,Nb,Mo)C ceramic was found at 15 ± 0.8 GPa, compressive ultimate strength—at 1.6 ± 0.1 GPa and fracture toughness—at 4.4 ± 0.1 MPa∙m^1/2^. The oxidation behavior of the produced ceramics in an oxygen-containing atmosphere was studied in the range of 25 to 1200 °C by means of high-temperature in situ diffraction. It was demonstrated that (Hf,Zr,Ti,Nb,Mo)C ceramics oxidation is a two-stage process accompanied by the change of oxide layer phase composition. As a possible mechanism of oxidation, diffusion of oxygen into the ceramic bulk results in the formation of a complex oxide layer made of c–(Zr,Hf,Ti,Nb)O_2_, m–(Zr,Hf)O_2_, Nb_2_Zr_6_O_17_ and (Ti,Nb)O_2_ was proposed.

## 1. Introduction

In that class, transition metals carbides may be highlighted, characterized by covalent, ionic covalent or metallic bonds affecting physicomechanical properties, including high hardness, strength, thermal conductivity, etc. Carbides are traditionally applied in cutting tools, friction units, catalysts, thermally-loaded constructional elements, etc. [1]. However, due to intense oxidation, the carbide systems’ main drawbacks are high embrittlement and low thermal stability in an oxygen-containing atmosphere. 

The entropy approach used in developing the novel multicomponent materials widened the class of ceramic materials. At the same time, various combinations of initial carbides provided the complex physicomechanical or thermal characteristics required for different applications. Multicomponent configurational entropy stabilized ceramics appear as a substitutional solid solution composed of three or more metals in an equimolar/non-equimolar ratio [2,3]. Due to the structurization features (mainly, stirring effect and distorted lattice resulting in delayed diffusion), these materials are characterized by an excellent complex of physicomechanical properties, including high hardness, fracture toughness and thermal stability [4,5,6]. The main factors affecting the formation of single-phase solid solution ceramics are lattice mismatch of the initial components and metal atomic size difference δ, which should be less than 6.6% [7,8,9,10]. The higher δ, the more obstructed the formation of a multi-phase solid solution. Therefore, carbides of groups IV-VBB metals are often used as initial components. They have similar crystal structures (face-centred cubic lattice, FCC), small differences in atomic radiuses and excellent mutual solubility. However, according to an entropy-forming-ability (EFA), descriptor developed for the prediction of the single-phase state of multicomponent ceramics [11,12], the possibility of single-phase ceramics production using the carbides of VI group metals with crystal lattices different from FCC having low solubility in carbides of metals of IV-VBB groups was experimentally confirmed. Introducing the carbides of metals of the VI group into multicomponent ceramic composition influences its properties. For example, Mo doping into an entropic solid solution results in densification due to relatively low melting temperature (2410 °C) and decreased grain size [13]. In addition, papers [8,14,15] showed that the system (TiZrHfTaMo)C was characterized by a combination of good plasticity and mechanical and tribological properties, which makes it a potential candidate for operation in tribocouples under high-speed friction. So, ceramics (Hf,Mo,Nb,Ta,Ti)C have good anti-wear properties at temperatures below 600 °C in tribological tests, and their wear resistance sharply deteriorates at 900 °C [14]. Since there is a local temperature rise in the tribocontact zone, it is important to know the behavior of the material during the temperature rise, including its phase stability. 

It is known that multicomponent ceramics possess high thermal stability compared to monocarbides owing to delayed diffusion. On the other hand, the mechanism of multicomponent ceramics oxidation is still poorly studied. It was shown in works [16,17,18,19,20,21] that the oxidation of multicomponent carbide systems follows a parabolic law in the temperature range from 800–1600 °C, which is associated with a diffusion-controlled mechanism of mass transfer in the oxidation process. The most studied ceramics is (TiZrNbTaHf)C. Backman et al. [22,23,24] showed that the preferential oxidation of each metal component in the (HfZrTaNbTi)C system was associated with the relative thermodynamic stability of their respective oxides. Group IV elements (Hf,Zr,Ti) showed the most favorable formation of oxides compared to Group V metals (Ta, Nb) because their oxides have the highest melting points and are the most thermodynamically preferable among refractory elements. The oxidation of Hf and Zr was more favorable than Ti, while Ta is preferably oxidized compared to Nb. According to Wang et al., the mechanism of Ti-containing multicomponent ceramics oxidation is controlled by the outward diffusion of the TiC-TiO active oxidation product [25]. It was also demonstrated that the inward diffusion of the oxidizing agent is the controlling stage during the oxidation [18,19,20,25]. However, there is a lack of data concerning the thermal stability of systems (Ti/Zr/Hf/Nb/Ta/Mo)C.

The present work investigated the oxidation behavior of (Hf,Zr,Ti,Nb,Mo)C ceramics in a 25–1200 °C air atmosphere temperature range.

## 2. Materials and Methods

The samples of (Hf,Zr,Ti,Nb,Mo)C solid solutions were studied. The commercially available TiC, ZrC, NbC, HfC and Mo_2_C powders (Izhevsk, Russia) were used as starting materials for producing high-entropy carbide ceramics. The starting metal carbide powders possessed the following phase composition: TiC, ZrC, NbC, and HfC had cubic lattices, and Mo_2_C had orthorhombic lattices. The parameters of the unit cell powders of metal carbides were: a(HfC) = 4.6259 Å, a(ZrC) = 4.6937 Å, a(TiC) = 4.3248 Å, a(NbC) = 4.4660 Å, Mo_2_C: a = 4.7333 Å, b = 6.0238 Å, c = 5.2103 Å. 

Equimolar TiC–ZrC–NbC–HfC–Mo_2_C powder systems were mixed in the planetary mill (AGO, Novosibirsk, Russia) equipped with steel drums with teflon inlet and alumina grinding bodies for 20 min in an argon atmosphere. The ceramic materials were produced by sintering at 1900 °C under 35 MPa pressure with isothermal soaking for 30 min with a 125 °C/min heating rate. 

The samples were prepared according to the standard technique of polished microspecimens preparation. X-ray diffraction analysis (XRD) was performed using an XRD-7000S diffractometer (Shimadzu, Kyoto, Japan) with Cu-Kα source (λ = 1.5405 Å). The oxidation behavior of the prepared ceramics was studied in an air atmosphere using high-temperature XRD in the temperature range from 25 to 1200 °C with the following parameters: 2θ from 24 to 41°, step angle of 0.0143°, time of exposure of 120 s, heating rate of 10 °C/min, capture step of 20 °C/image. The phase composition was identified using Match! Software (Crystal Impact, Bonn, Germany) and Crystallography Open Database (COD).

Lattice distortion *δ* was determined by the Formula (1) [8]:

(1) $$\delta =\sqrt{\sum_{i=1}^{n}c_{i}{\left(1-\frac{a_{i}}{\sum_{i=1}^{n}c_{i}a_{i}}\right)}^{2}},$$

where *c_i_* is the atomic percentage, *a_i_* is the lattice parameters of the *i*-th component. 

Thermal analysis was performed on STA 449 F1 Jupiter instrument (Netzsch, Germany) in the temperature range from 50 to 1200 °C with a heating rate of 10 °C/min in airflow.

The Microstructure of the prepared samples was investigated using scanning electron microscopy (SEM) on «LEO EVO 50» microscope (Zeiss, Jena, Germany). Next, the distribution of chemical elements was analyzed using energy-dispersive spectroscopy (EDS) (Inca x-ACT, Oxford Instruments Analytical, Oxford, UK). Finally, the density (*ρ*) of the prepared samples was determined using the hydrostatic weighting method.

The hardness of the pre-polished samples was measured on AXIOVERT-200MAT (Zeiss, Jena, Germany) metallographic microscope by indentation with a Wickers pyramid under 1000 gs load for 10 s. The strength of the produced samples was studied using Instron—1185 testing machine (Instron, Norwood, MA, USA). A compression test was performed at a load speed of 0.05 mm/s using INSTRON—1185 testing machine (Instron, Norwood, MA, USA). The strength of the brick-shaped samples under axial compression was calculated according to Formula (2):

(2) $$\sigma =\frac{P}{S},$$

where *P* is the critical load, *S* is the area under load. 

Fracture toughness was measured by the V-type notch method with accordance to the ISO 23146:2008 standard according to the Formula (3):

(3) $$K_{IC}=f\left(\frac{P_{max}L10^{-6}}{bW^{3/2}}\right)\left(\frac{3{\left[a/W\right]}^{1/2}}{2{\left[1-a/W\right]}^{3/2}}\right),$$

where $f=\frac{1.99-\left[a/W\right]\left[1-a/W\right]\left[2.15-3.93\left[a/W\right]+2.7{\left[a/W\right]}^{2}\right]}{1+2\left[a/W\right]}$, *L*—distance between the lower support, mm; *b*—sample width, mm; *W*—sample height, mm; *a*—depth of V-type notch, mm.

## 3. Results

### 3.1. (Hf,Zr,Ti,Nb,Mo)C Multicomponent Carbide Ceramic

It is known that the formation of a single-phase solid solution is significantly affected by the chemical compatibility of the elements, i.e., the details must satisfy the Hume-Rothery rule (*δ*) for solid solutions of substitution. The mismatch between the lattice parameters of the initial components plays an important role in forming a homogeneous single-phase state [10]. Calculations showed that the lattice mismatch was 3.33%, corresponding to the criterion δ ≥ 6.6% for forming a monophase state of highly entropic materials [7,8,9,10]. Generally, the higher the discrepancy in the unit cell sizes, the more unfavorable the conditions for the formation of solid solutions. Thus, in the system, HfC-ZrC-TiC-NbC-Mo_2_C can form a homogeneous solid solution (Hf,Zr,Ti,Nb,Mo)C.

**Figure 1:**
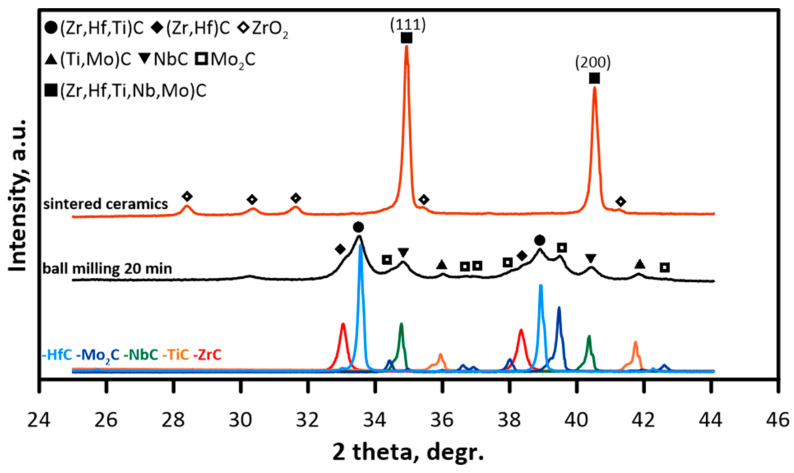
X-ray diffractograms of the initial powder mixture, powder mixture after 20 min of mechanical treatment and (Hf,Zr,Ti,Nb,Mo)C sintered ceramic.

The diffractograms of the initial HfC–ZrC–TiC–NbC–Mo_2_C powder mixture after mechanical treatment in the planetary mill are presented in Figure 1. The characteristic reflexes of HfC, ZrC, TiC, NbC and Mo_2_C monocarbides were observed on the diffractogram of the initial powder mixture. On the diffractogram of the powder mixture mechanically treated for 20 min, the shift and broadening of the reflexes corresponding to initial carbides and the changes in their intensity were observed. During the milling process accompanied by mechanical treatment, the reflex’s decreased intensity and broadening may be attributed to crystallite size reduction, crystal structure refinement and lattice deformations. On the diffractogram of the mechanically treated powder mixture, the reflexes specific for Zr-enriched (Hf,Zr)C solid solution, Mo-enriched (Ti,Mo)C and (Hf,Zr,Ti)C with additional cation depletion in NbC, Mo_2_C carbides.

The sintering of TiC–ZrC–NbC–HfC–Mo_2_C powder mixtures mechanically treated for 20 min at 1900 °C under the pressure of 35 MPa resulted in the formation of single-phase (Hf,Zr,Ti,Nb,Mo)C solid solution with FCC lattice. The cell dimension was found at 4.4524 Å. The size of the coherent diffracting domain calculated using the Scherrer formula was found at 60 nm, micro distortion <ε>—at 1.4 × 10^−3^. The content of the oxide phase calculated as a ratio of the total intensity of the oxide’s reflexes to the total intensity of all reflexes was found at 8%. 

After the mechanical treatment of the powder mixture, diffraction lines corresponding to oxide phases were observed, which may be due to the condition of the initial powders as well as partial oxidation during the technological processes. Traditionally, metal carbide powders are produced by carbonizing corresponding oxides in carbon-containing flow. For that reason, metal carbides usually contain a small amount of residual oxygen [26,27]. It is known that oxygen impurities (e.g., ZrO_2_) are always present on the surface of ZrC particles, and oxygen atoms may substitute carbon atoms in ZrC crystal lattice, forming oxycarbides solid solutions (ZrC_x_O_y_) [28]. The reaction product is the solution of oxygen in the ZrC phase (lattice oxygen) rather than the separate oxycarbide phase ZrC_x_O_y_.

The density of sintered (Hf,Zr,Ti,Nb,Mo)C ceramic, measured using hydrodynamic weighting, was 7.76 ± 0.03 g/cm^3^. The theoretical density of the solid solution was calculated according to the Formula (4):

(4) $$\rho_{th}=\frac{1.6605\cdot 4({0.1667(M}_{Nb}+M_{Ti}+M_{Zr}+M_{Hf})+{0.3333M}_{Mo})+M_{c})}{a^{3}},$$

where *M* is the molar mass of the element, g/mol, *a*—crystal lattice parameter, Å. The theoretical density of the sintered ceramic was 8.46 g/cm^3^, while the relative density was 92%. The porosity determined by the Formula,

(5) $$\theta =\left(1-\frac{\rho_{g}}{\rho_{th}}\right)100\%$$

where *ρ_g_*-hydrostatic density, *ρ_th_*_—_theoretical density, was 8%. It should be noted that the presence of porosity is a positive factor for the thermal resistance of ceramic materials because the pore space restrains the incipient microcracks in the process of thermal effect, formed due to the mismatch of thermal expansion coefficients of carbide and oxidation products, and inhibits their distribution throughout the material.

**Figure 2:**
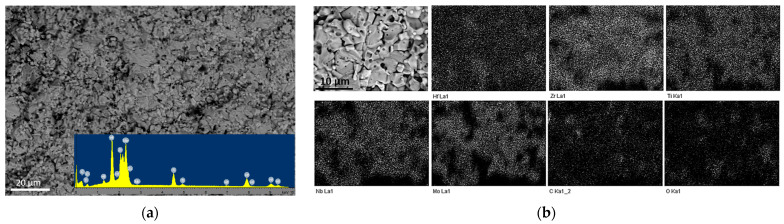
Fracture surface (**a**) and elemental mapping (**b**) of ceramics (Hf,Zr,Ti,Nb,Mo)C.

Figure 2 shows the SEM image of the (Hf,Zr,Ti,Nb,Mo)C fracture and the corresponding EDS mapping. It can be seen that the microstructure is represented by large grains with an average size of 4.8 ± 2.1 µm and small grains d = 1 ± 0.4 µm. The average grain size of the (Hf,Zr,Ti,Nb,Mo)C ceramic measured using the random linear intercept method from fracture microimages was found at 3.8 ± 2.4 µm. The calculated values of ceramic (Hf,Zr,Ti,Nb,Mo)C grain sizes are comparable or lower than analogous values for ceramics (TiZrHfNbTaMo)C [29] synthesized by non-pressure sintering at the temperatures from 2200 to 2500 °C (2.8 ± 1.3 and 15.2 ± 6.5 µm, respectively), (TiZrHfNbTaMo)C [14] obtained by SPS at temperatures from 1950 to 2050 °C in steps of 50 °C (1.7 ± 0.6 and 5.2 ± 1.6 µm, respectively) and lower by comparison with the results of microstructure studies (TiZrNbTaMo)C [13], obtained by two-stage hot pressing (8.8 ± 3.0 µm). According to the results of EDS analysis of the fracture (Hf,Zr,Ti,Nb,Mo)C solid solution surface, the atomic concentration of the chemical elements was found as the following: C—56.11 at.%, O—13.52 at.%, Ti—4.53 at.%, Zr—6.43 at.%, Nb—5.04 at.%, Hf—5.62 at.%, Mo—8.85 at.%. The concentration of molybdenum was higher than other metal elements due to its excess in the form of Mo_2_C in the initial powder mixture. The results confirm the mutual diffusion of elements by forming a homogeneous solid solution. 

The hardness of (Hf,Zr,Ti,Nb,Mo)C ceramic was found at 15 ± 0.8 GPa, compressive ultimate strength—at 1.6 ± 0.1 GPa and fracture toughness—at 4.4 ± 0.1 MPa∙m^1/2^. The obtained hardness values are comparable or lower in comparison with the hardness of high-entropic carbides of similar composition whose values ranged from 15 GPa for the system (TiZrHfNbTaMo)C [14] to 25.45 GPa for (TiZrNbTaMo)C_0.85_ [30]. At the same time, the fracture toughness value exceeds the known data, which were 3.7 MPa∙m^1/2^ for (TiZrHfNbTaMo)C [29] and 4.2 MPa∙m^1/2^ for (TiZrHfNbTaMo)C [14]. The mechanical properties of carbide ceramics depend on Me_x_C_y_ stoichiometry, porosity, grain size and oxygen in the lattice and oxides in the composition. Therefore, the low hardness and strength values are probably due to porosity and the presence of oxide phases in the composition of ceramic samples (Hf,Zr,Ti,Nb,Mo)C. 

### 3.2. The Study of Structural and Phase Condition of Multicomponent Ceramics under High-Temperature Conditions

**Figure 3:**
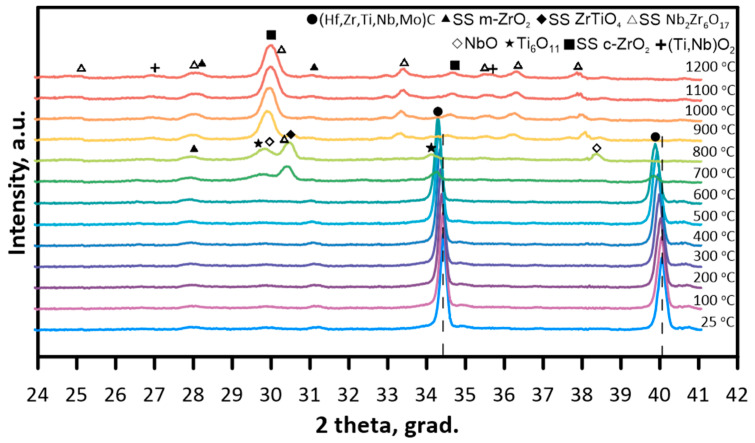
Diffractograms of (Hf,Zr,Ti,Nb,Mo)C sintered ceramics during the heating from 25 to 1200 °C.

According to the results of high-temperature in situ X-ray diffraction analysis in the range from 25 to 1200 °C, the reflexes corresponding to (111) and (200) lattices of (Hf,Zr,Ti,Nb,Mo)C solid solution were observed (Figure 3). At a temperature higher than 700 °C, the decrease of solid solutions reflexes intensity, and the appearance of wide oxide reflexes were observed. Due to the diffraction lines overlapping and possible ion replacement of metal cations in the oxide lattices, qualitative analysis was complicated. On the diffractograms of the ceramics heated up to 700 °C, the reflexes of solid solutions based on ZrTiO_4_, Nb_2_Zr_6_O_17_, m-ZrO_2_, titanium and niobium oxides. Further decrease of temperature up to 900 °C resulted in the changes in phase composition. The most intensive reflexes were attributed to cubic ZrO_2_-based solid solution. In addition, the appearance of Nb_2_Zr_6_O_17_ reflexes was observed, which confirms the increase in the content of that phase. (Ti,Nb)O_2_ solid solution reflexes were also detected. According to the results of papers [31,32], the carbon formed by the oxidation of ZrC carbide stabilizes c-ZrO_2_ at sufficiently low temperatures.

**Figure 4:**
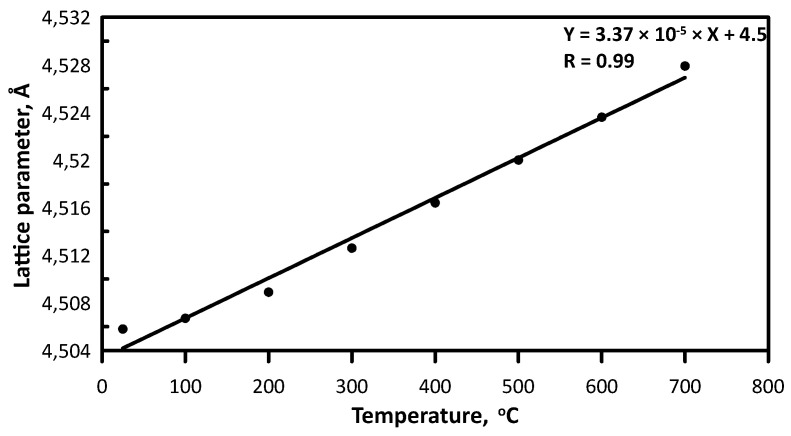
Lattice parameter of the sintered (Hf,Zr,Ti,Nb,Mo)C ceramic at versus temperature.

In the range from 25 to 600 °C the diffractograms of (Hf,Zr,Ti,Nb,Mo)C solid solution were nearly identical. However, with the increase in temperature, the reflexes became more asymmetric, and the shift of (Hf,Zr,Ti,Nb,Mo)C reflexes to lower angles was observed, which demonstrates the growth of the crystal lattice parameter. Cell dimensions were calculated from the (111) and (200) reflexes according to the Formula

(6) $$\frac{1}{d^{2}}=\frac{h^{2}+k^{2}+l^{2}}{a^{2}}$$

due to the limited angle range of the high-temperature X-ray diffraction. It was revealed that with the increase of temperature from 25 to 700 °C the cell dimension was increased from 4.5058 to 4.5279 Å (Figure 4), which may be a result of several factors, including insignificant lattice distortion due to the thermal atomic rearrangement and thermal expansion of the studied ceramic. Thermal expansion coefficient (CTE) calculated according to the Formula,

(7) $$\alpha =\frac{\Delta a}{a_{0}\cdot \Delta T}$$

where *a*_0_ is the value of the lattice parameter at room temperature, Δ*a* is the variation of the lattice parameter at a temperature variation Δ*T* from 25 to 700 °C, was found at 7.3 × 10^−6^ °C^−1^. The calculated value insignificantly exceeds CTE predicted using the rule of mixtures and the values for the components: HfC (6.58 × 10^−6^ °C^−1^), ZrC (6.25 × 10^−6^ °C^−1^), TiC (7.23–7.31 × 10^−6^ °C^−1^), NbC (6.45–6.76 × 10^−6^ °C^−1^), Mo_2_C (α_a_ = 4.8 × 10^−6^ °C^−1^, α_c_ = 8.7 × 10^−6^ °C^−1^) [33,34], which was found at 6.26–7.12 × 10^−6^ °C^−1^ with Mo_2_O anisotropy taken into account. According to the previous calculations, the CTE of (TiZrHfTaMo)C is comparable to one of HfC (7.86 × 10^−6^ °C^−1^) and (TiZrHfTaNb)C (7.74 × 10^−6^ °C^−1^) [8].

The reflexes of (Hf,Zr,Ti,Nb,Mo)C solid solution were not observed at temperatures higher than 745 °C. In the temperature range from 25 to 600 °C, the size of (Hf,Zr,Ti,Nb,Mo)C solid solution coherent diffracting domain was around 63 nm, while at 700 and 725 °C–50 and 30 nm, respectively. 

**Figure 5:**
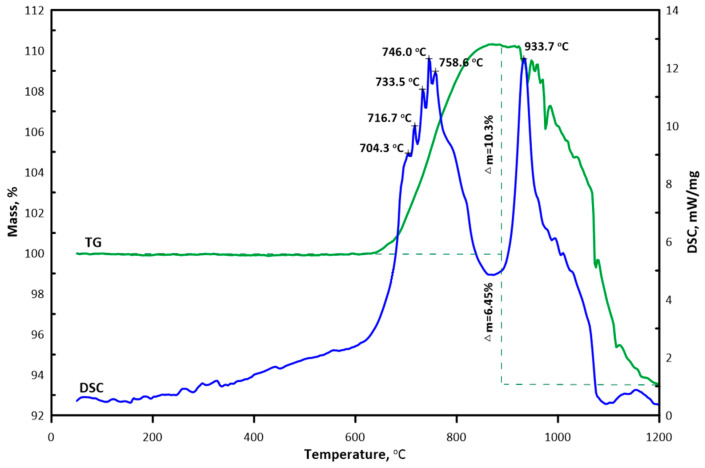
TG-DSC curves of (Hf,Zr,Ti,Nb,Mo)C ceramic in the 25–1200 °C range.

TG-DSC results demonstrated that (Hf,Zr,Ti,Nb,Mo)C solid solution was stable in air at a temperature up to 673 °C with subsequent surface oxidation of the ceramic (Figure 5). Several exothermic peaks (704, 717, 734, 746 and 759 °C) corresponding to the oxides formed on the ceramic surface appeared with further temperature increase. Mass gain at 900 °C was found to be 10.3%. The thermal effect at 934 °C was attributed to 6.45% mass loss. Atypical behavior of the TG line higher than 934 °C (particularly, the appearance of several peaks) may be explained by the release of volatile compounds with the formation of new surfaces, their further oxidation and, as a result, insignificant uneven changes in the sample mass.

**Figure 6:**
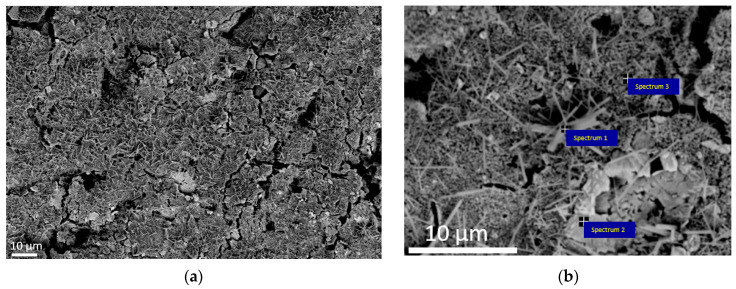
Microstructure of the oxide layer formed on the surface of (Hf,Zr,Ti,Nb,Mo)C ceramic after high-temperature treatment at 1200 °C at various magnifications: (**a**) ×2000; (**b**) ×10,000.

The oxide layer formed on the surface of (Hf,Zr,Ti,Nb,Mo)C ceramic after the high-temperature diffraction was presented by grains of various morphologies, including small oxide phase nuclei with a spherical shape, individual crystals with elongated and irregular shape (Figure 6a). EDS analysis demonstrated the following content of metal elements: Nb (8.01 at.%), Ti (4.98 at.%), Zr (4.98 at %), Hf (3.19 at.%), Mo (1.01 at.%) (Table 1). Detailed analysis showed that the elongated crystals contained O, Ti, Nb and Hf atoms. Moreover, niobium content was two-fold higher than the other elements (Figure 6b). Irregular-shaped crystals had Zr, Hf and O, corresponding to (Zr,Hf)_x_O_y_ solid solution. The area of small nuclei contained O, Ti, Zr, Nb and Hf with that, hafnium depletion was observed compared to other metals. Compared to EDS and XRD, results suggest that the formed solid solution had a composition of (Zr,Hf,Ti,Nb)O_2_. Atomic concentrations of the chemical elements are presented in Table 1.

**Figure 7:**
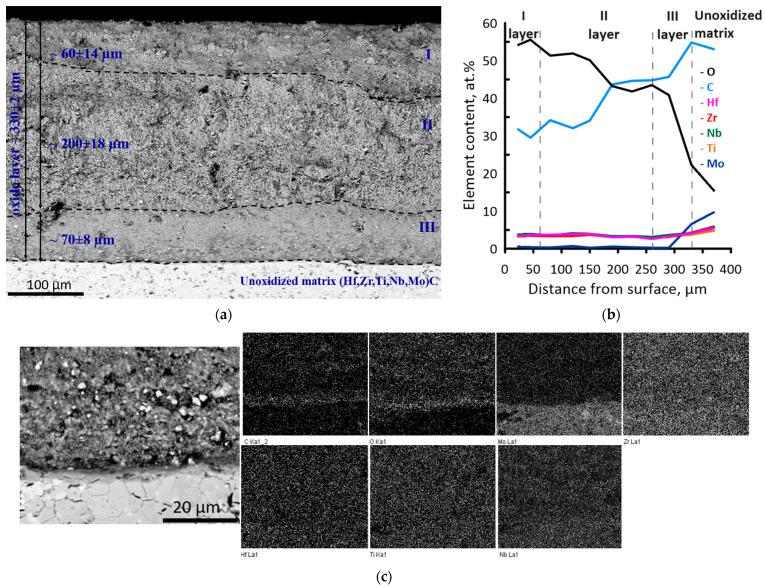
SEM image of (**a**) cross-section of (Hf,Zr,Ti,Nb,Mo)C ceramic after high-temperature treatment at 1200 °C; (**b**) atomic concentration of the elements on the prepared cross-section; (**c**) EDS mapping on the “matrix-oxide” interface.

A cross-section of (Hf,Zr,Ti,Nb,Mo)C ceramic after high-temperature treatment at 1200 °C is presented in Figure 7a. It was observed that after the high-temperature treatment, an oxide layer with lamellar structure, various microstructures and porosity was formed: a higher dense layer (~60 µm thickness) with large individual pores, porous layer with dendrite-like microstructure (~200 µm thickness) and lower dense layer (~70 µm) with residual porosity. The total thickness of the oxide layer was 330 ± 2 µm. Elemental analysis with a 30 µm scanning step from the outer layer of the oxidized surface to the unoxidized matrix revealed that Mo content in the oxide layer did not exceed 1 at.%, while the content of other metal elements was nearly equal and was found around 4 at.% (Figure 7b). The “Ceramic matrix-oxide layer” interface was well-defined, but the crack formation was not observed. From the EDS data, a thin O- and C-rich layer was formed on the “ceramic matrix-oxide layer” interface, Figure 7c. Single-point EDS analysis revealed that the white grains near the interface contained C, O, Ti, Zr and Hf with significant Ti depletion. Grey highly-dispersive grains contained all metal components in nearly equal proportion.

## 4. Discussion

Combined interpretation of high-temperature diffraction and thermal analysis suggests high stability of (Hf,Zr,Ti,Nb,Mo)C ceramic system in the range from 25 to 673 °C. Thermal effects in 700–760 °C interval correspond to the ceramic oxidation followed by the formation of complex oxides based on ZrTiO_4_, Nb_2_Zr_6_O_17_, m-ZrO_2_, Ti_x_O_y_ and Nb_x_O_y_, which is confirmed by XRD results. In work [21], it is shown that an oxide layer containing a mixture of complex oxides, including 4-metal oxides, was formed on the surface of the studied ceramics during the oxidation process in the air. As expected, the oxidation process was accompanied by mass gain of the ceramic sample. The thermal effect with a maximum of 934 °C is attributed to forming (Zr,Hf,Ti,Nb)O_2_ solid solution and the original phases. Energy dispersive spectroscopy of the cross-section of the oxide layer formed on the surface of (Hf,Zr,Ti,Nb,Mo)C ceramic revealed the formation of C- and O-rich interlayer and molybdenum depletion. It is known that carbide oxidation proceeds via the formation of oxycarbides (MeC_1−x_O_x_) [35]. With the temperature increase, oxygen solubility in the lattice increases resulting in oxycarbide lattice rearrangement with the formation of an oxide lattice. When the oxygen content in the MeC_1−x_O_x_ system is close to x∼0.4, the oxycarbide degrades to oxide and carbon. On that step, metal is oxidized with the formation of the oxide layer, which acts as a barrier for oxygen diffusion. Released carbon does not oxidize due to the low local activity of oxygen and remains on the interface. Thermal stress, which results from different CTE of the matrix and formed oxides, leads to the formation of cracks followed by oxygen permeation and oxidation of carbon with the release of C_x_O_y(g)_ gaseous products resulting in porosity of the oxide layer.

Charpentier et al. [31,36] demonstrated that such an interlayer reduces the oxidation process as the diffusion coefficient of oxycarbides is much less than oxides. Molybdenum deficiency in the oxide layer results from releasing volatile non-stoichiometric molybdenum oxides [37]. The family of molybdenum suboxides is well known. Among them, MoO_2_ and MoO_3_ are the most stable. With that, MoO_3_ is the most volatile compound. The process of MoO_3_ sublimation takes place at 800 °C, and with every 50 °C of temperature increase sublimation rate rises by an order, which explains high Mo losses [38]. Mass loss of (Hf,Zr,Ti,Nb,Mo)C ceramic observed around 934 °C is most likely due to the release of Mo_x_O_y(g)_ and C_x_O_y(g)_ oxidation products. 

The enhanced oxidation resistance of multicomponent ceramics is mainly explained by the formation of oxide systems on their surface during oxidation and further reduced diffusion of metal cations. The diffusion rate of the entropy solid solution is limited by the lowest diffusion rate of the metal element in its composition. For the (Hf,Zr,Ti,Nb,Mo)C ceramic, metal elements in TiC and Mo_2_C demonstrate the lowest diffusion rate. However, the developed porous structure of the oxide layer, formed during the release of gaseous products, intensifies the material oxidation due to the enhanced oxygen diffusion to the unoxidized ceramic matrix. 

It was previously demonstrated [25] that the oxidation of Ti-containing multicomponent ceramics is controlled by the outward diffusion of TiC–TiO active oxidation product, which is being additionally oxidized to TiO_2_, reacts with the other oxides with the formation of complex oxide systems hindering oxygen migration in the carbide matrix. According to the results of layer-by-layer EDS, titanium depletion/enrichment was not observed, and the atomic ratio of metal elements was found to be equal (except for molybdenum). The obtained results demonstrate that, most probably, in (Hf,Zr,Ti,Nb,Mo)C ceramic system, the oxidation process is controlled by the oxygen diffusion to the “ceramic matrix-oxide layer” interface than metal cations diffusion to the outer oxide layer.

## 5. Conclusions

Single-phase ceramic solid solution (Hf,Zr,Ti,Nb,Mo)C with HCC structure was obtained by sintering under pressure. In addition, the structure, and the oxide behavior in the heating process from 25 to 1200 °C in the air were studied. Based on the obtained data, the following conclusions can be made:

(1) It is shown that during the mechanical processing of equimolar powder mixture of TiC–ZrC–NbC–HfC–Mo_2_C carbides, the formation of double and triple solid solutions occurs, which facilitates the formation of single-phase state (Hf,Zr,Ti,Nb,Mo)C in the sintering process.
(2) The average ceramic (Hf,Zr,Ti,Nb,Mo)C grain size was equal at 3.8 ± 2.4 µm. The CTE was 7.3 × 10^−6^ °C^−1^ (25–700 °C), the hardness was found at 15 ± 0.8 GPa, the compressive strength was equal at 1.6 ± 0.1 GPa, the fracture toughness was seen at 4.4 ± 0.1 MPa∙m^1/2^.
(3) A study of the oxide behavior of ceramic (Hf,Zr,Ti,Nb,Mo)C in the temperature range 25–1200 °C showed that the ceramic system (Hf,Zr,Ti,Nb,Mo)C remains stable when the temperature rises from 25 to 673 °C followed by two-stage oxidation accompanied by changes in oxidation products and mass of ceramic samples.
(4) The microstructure of the oxidized surface of ceramic (Hf,Zr,Ti,Nb,Mo)C was studied. The temperature exposure on the sample’s surface was determined to form a layered structure with different microstructures and porosity. Furthermore, the oxygen diffusion into the ceramic material volume leads to the formation of an oxide layer of complex composition c–(Zr,Hf,Ti,Nb)O_2_, m–(Zr,Hf)O_2_, Nb_2_Zr_6_O_17_ and (Ti,Nb)O_2_ was suggested as a possible oxidation mechanism.

## Figures and Tables

**Table 1:** The atomic concentration of the chemical elements on (Hf,Zr,Ti,Nb,Mo)C oxidized surface.

Spectrum	Element Content, at %					
	O	Hf	Zr	Ti	Nb	Mo
Integral	76.1	3.2	5.0	6.7	8.0	1.0
1	84.4	1.5	5.0	4.3	4.8	–
2	86.5	2.5	11.0	–	–	–
3	77.2	4.0	–	6.0	12.8	–

## Data Availability

Not applicable.
